# Biomechanical comparison of a C1 posterior arch clamp with C1 lateral mass screws in constructs for C1-C2 fusion

**DOI:** 10.1177/09544119211032479

**Published:** 2021-07-19

**Authors:** Timothy L Lasswell, John B Medley, Jack P Callaghan, Duane S Cronin, Colin D McKinnon, Supriya Singh, Parham Rasoulinejad

**Affiliations:** 1 Department of Mechanical and Mechatronics Engineering, University of Waterloo, Waterloo, ON, Canada; 2 Department of Kinesiology, University of Waterloo, Waterloo, ON, Canada; 3 Division of Orthpaedic Surgery, Department of Surgery, Western University and Victoria Hospital, London Health Sciences Centre, London, ON, Canada

**Keywords:** Spinal implants, biomechanical testing/analysis, spine biomechanics, biomedical devices, implants/prosthetics, orthopaedic procedures

## Abstract

The aim of this experimental study was to assess the biomechanical performance of a novel C1 posterior arch (C1PA) clamp compared with C1 lateral mass (C1LM) screws in constructs used to treat atlantoaxial instability. These constructs had either C2 pedicle (C2P) screws or C2 translaminar (C2TL) screws. Eight fresh-frozen human cadaveric ligamentous spine specimens (C0-C3) were tested under six conditions: the intact state, the destabilized state after a simulated odontoid fracture, and when instrumented with four constructs (C1LM-C2P, C1LM-C2TL, C1PA-C2P, C1PA-C2TL). Each specimen was tested in a spinal loading simulator that separately applied axial rotation, flexion-extension and lateral bending. In each test condition, displacement controlled angular motion was applied in both directions at a speed of 2 deg/s until a resulting moment of 1.5 Nm was achieved. The measured ranges of motion (ROM) of the C1-C2 segments were compared for each test condition using nonparametric Friedman tests. The destabilized state had significantly more C1-C2 motion (*p* < 0.05) than the intact state in all cases, and all constructs greatly reduced this motion. C2 pedicle screw constructs that used the C1PA clamp had significantly less C1-C2 motion (*p* < 0.05) than those with C1LM screws in flexion-extension as well as axial rotation and no statistically significant difference was detected in lateral bending. C2 translaminar screw constructs that used the C1PA clamp had significantly less C1-C2 motion (*p* < 0.05) than those with C1LM screws in flexion-extension and no statistically significant difference was detected in axial rotation or in lateral bending. Data from the current study suggested that constructs using the novel C1PA clamp would provide as good, or improved, biomechanical stability to the C1-C2 segment compared with constructs using C1LM screws.

## Introduction

The upper cervical spine has large ranges of motion (ROM), particularly in axial rotation and flexion-extension that are controlled by ligaments with considerable laxity.^1^ Thus, when a Type II odontoid fracture^2^ occurs, there is a substantial and life threatening destabilization of the upper cervical spine. Type II odontoid fractures have been reported as the most prevalent spine injury in the octogenarian population.^3^

Operative vs. non-operative management of these fractures in the geriatric patient population is a controversial topic. For both approaches in octogenarians, Graffeo et al.^4^ reported the same mortality of 41% at 1-year. However, Iyer et al.^5^ published a review to support the idea that most octogenarians were better managed non-operatively whereas Faure et al.^6^ promoted operative management with posterior instrumented fusion. Although the short-term risks of non-operative treatments were considered low, the resulting nonunion rates were high^7^ and could be improved to nearly 100% by performing a C1-C2 fusion.^8^

Posterior instrumented fusion has been described as the most popular surgical technique^8^ and most commonly involves a construct with C1 lateral mass (C1LM) screws connected to C2 pedicle (C2P) screws with rods. This technique was pioneered by Goel et al.^9^ and modified by Harms and Melcher.^10^ It was recently promoted by Faure et al.^6^ but they did admit that it was demanding and that the main technical challenge was related to bleeding of the venous plexus around the C2 nerve root and at the screw entry points on the C1 lateral masses. Huang et al.^8^ also expressed concerns regarding the risk of blood loss during exposure of the C1 lateral masses and noted the additional intraoperative risks of vertebral artery injury and nerve dysfunction after dissection of the C2 nerve root. Thus, morbidity for operative techniques remained high.^4^

Some benefit was claimed by Wright^11^ for using C2 translaminar (C2TL) screws that offered a C2 fixation method that mitigated the risk of vertebral artery injuries while still achieving excellent fusion rates.^12^ However, with the continued use of C1LM screws, many of the previously mentioned risks remained.

A claw for fixation at the C1 posterior arch was proposed by Olerud and Olerud^13^ but this device was intended for use with bilateral C1-C2 transarticular screws that were technically challenging to place. In a cadaveric study by Henriques et al.,^14^ eight human cervical specimens (C0-C3) were loaded non-destructively in a spinal motion simulator in order to assess the biomechanics of constructs that used the C1 claw. Constructs were tested under axial rotation, flexion-extension and lateral bending up to a load of 1.5 Nm. An axial pre-load of 50 N was added when testing axial rotation. Motion was captured using an optical motion analysis system (Optotrak; Northern Digital, Waterloo, ON) and ROM data at the C1-C2 segment was reported for each construct. One of the constructs investigated the biomechanics of the C1 claw when used with C2 pedicle screws but this construct did not achieve adequate C1-C2 segment stability under axial rotation and lateral bending.

Another alternative fixation method at C1 was explored by Kelly et al.,^15^ who proposed a plate attached to the C1 posterior arch with locking screws that was then connected to C2TL screws with rods. Biomechanical testing of this locking plate was performed in a cadaveric study using seven human cervical specimens (C0-C4) that were loaded non-destructively in a spinal motion simulator. Constructs were tested under axial rotation, flexion-extension and lateral bending up to a load of 1.5 Nm and an axial pre-load was not used. ROM data at the C1-C2 segment was reported for each construct. Statistical analysis of the C1 posterior arch locking plate construct connected to C2TL screws did not detect any significant differences in C1-C2 ROM compared with a construct using C1LM screws connected to C2TL screws, using rods. This suggested that fixation at the C1 posterior arch could provide adequate construct stability to achieve fusion while eliminating the risks associated with C1 lateral mass screws.

The current study investigates a method of posterior arch fixation consisting of a C1 posterior arch (C1PA) clamp connected to C2P screws, or C2TL screws, as another potential alternative to C1LM screws. Funding for this study was provided exclusively by The Natural Sciences and Engineering Research Council (NSERC) of Canada and the Ontario Government (OGS/QEII-GSST). After completion of this study, a United States patent was obtained to protect the novel aspects of the C1PA clamp. After the patent was issued by the United States Patent Office, it was assigned to Spinal Simplicity LLC (Overland Park, KS), a medical device company that has plans to commercialize the C1PA clamp. No funding was obtained from Spinal Simplicity for this study and they were not involved with the C1PA clamp until after completion of the present study.

**Figure 1.**
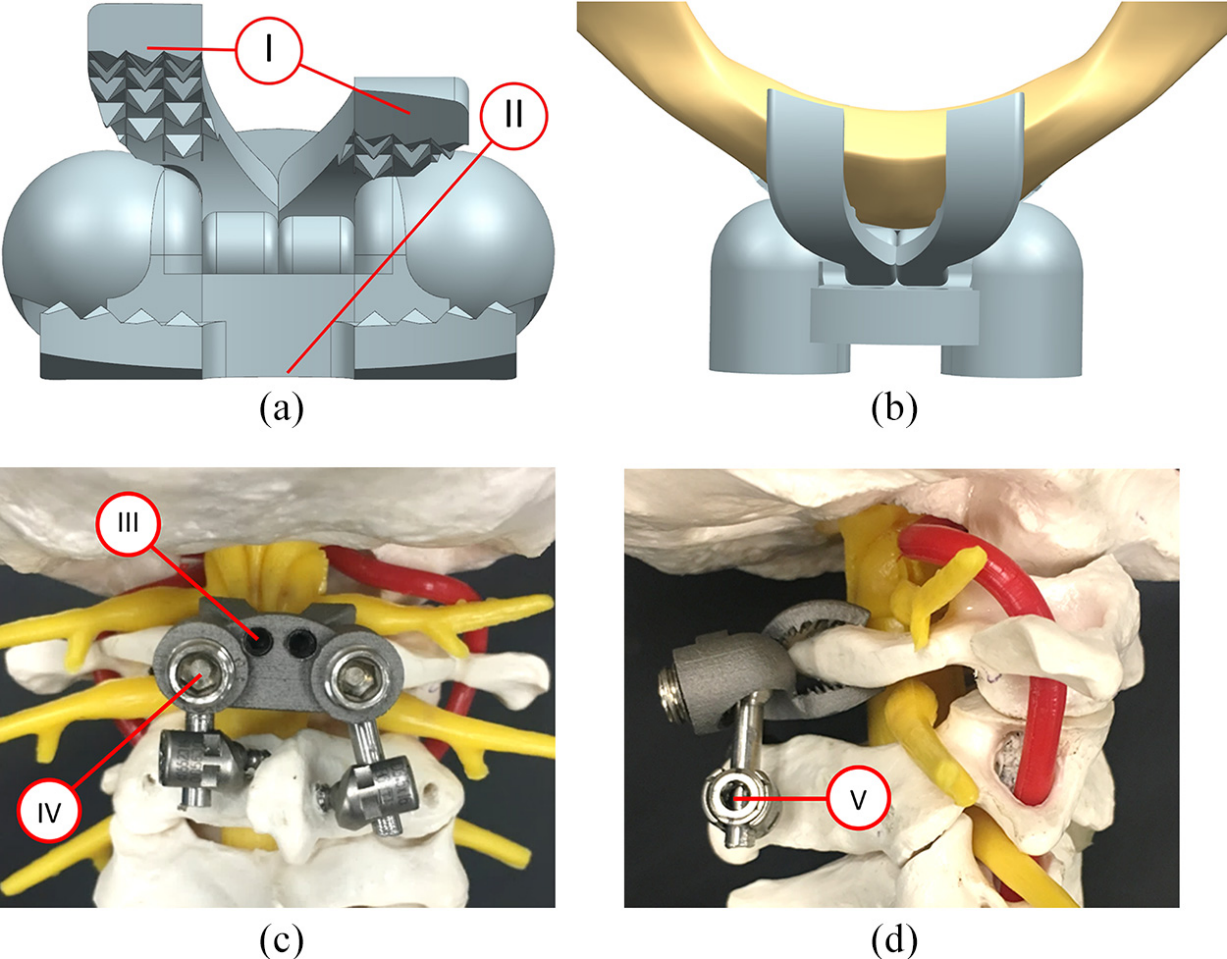
Computer renditions of the C1PA (with part numbers I, II, III, IV and V identified) showing (a) independent superior jaws and (b) axial view positioning along with a prototype clamp implant using C2TL crews attached to a polymeric spine model showing (c) posterior and (d) lateral views.

An implant, with two superior jaws, was designed to clamp to the posterior arch of C1 as a part of a C1-C2 construct (Figure 1(a)). There was some intent for its various features but absolute proof of their abilities to perform as intended remain topics for future investigations. So, independent articulation of the superior jaws (Figure 1(a): part I) allowed for better fixation compared with a larger single jaw since the height of the posterior arch was often non-symmetrical about the midline. The lateral width of the superior jaws was limited to 20 mm centred about the midline to avoid potential injury to the vertebral artery.^16^ Since injury to the vertebral artery was considered to be less of a concern for the inferior jaw, the lateral width was 25 mm, centred about the midline, to further improve fixation. The inferior jaw contained a central cutout to provide a bone graft window that was intended to improve the quality of the long term fusion (Figure 1(a): part II). In the axial plane, the jaws were designed to have a curved profile so that the implant did not protrude into the spinal canal and risk injury to the dura or spinal cord (Figure 1(b)). After placement on the posterior arch, the C1PA clamp could be locked in place by tightening jaw locking set screws (Figure 1(c): part III) that acted as cam mechanisms to progressively close the jaws until adequate fixation was judged to be achieved. Fixation strength between the C1PA clamp jaws and the posterior arch was increased through the use of pyramid spikes with 1 mm heights. Polyaxial rods were then inserted into the sockets of the C1PA clamp and connected to the polyaxial screws used in C2. These polyaxial rods were locked into place by tightening the rod cap screws to 4 Nm (Figure 1(c): part IV) and the polyaxial set screws (Figure 1(d): part V), creating the C1-C2 construct.

It was hypothesized that no significant difference would be detected in the instrumented C1-C2 ROM when comparing constructs that used the C1PA clamp with constructs that used C1LM screws.

## Materials and methods

### Prototype fabrication

C1PA clamp prototypes were created using selective laser melting additive manufacturing by a Renishaw AM 400 system (ADEISS, London ON). Titanium allow powder (Ti6Al4V Grade 5) with a particle size of 15–45 µm was used as the raw material. Process parameters were set to a layer thickness of 40 µm and a 400 W laser with a spot size of 70 µm was used. A post-processing heat treatment was performed under an argon environment with the following schedule: heat from room temperature to 350°C taking 60 min, hold at 350°C for 30 min, heat from 350°C to 850°C taking 60 min, hold at 850°C for 60 min, cool to room temperature over 24 h. Implant sizing was determined by reviewing the C1 posterior arch height (in the sagittal plane) and width (anterior-posterior distance) of eight cadaveric specimens from CT scans. Based on height and width measurements of the posterior arch obtained from CT imaging, a single implant size was considered to be adequate for all of the C1 posterior arch anatomies present in the current study. These prototypes were for cadaveric testing purposes only since the C1PA clamp did not have any regulatory approval.

### Biomechanical testing

Eight fresh frozen cadaveric specimens containing the C0-C3 vertebrae were obtained (Science Care, Phoenix AZ) and ethics approval was granted for this study. The specimens comprised of six male specimens and two female specimens with an average age of 76.5 years (range: 69–85 years). All specimens were thawed and denuded in one day by two fellowship trained spine surgeons (authors SS, PR). This denuding involved removal of tissue until only the bony anatomy and ligamentous structures were intact. After denuding, the specimens had pilot holes drilled by the spine surgeons for the various polyaxial screws that were to be inserted when tests with the various constructs were conducted. This allowed testing to be done without needing the presence of the spine surgeons to place the constructs. After this preparation, the specimens were wrapped in 0.9% saline-soaked towels, placed in double-sealed plastic bags, and refrozen at −20°C until the testing dates.

**Figure 2.**
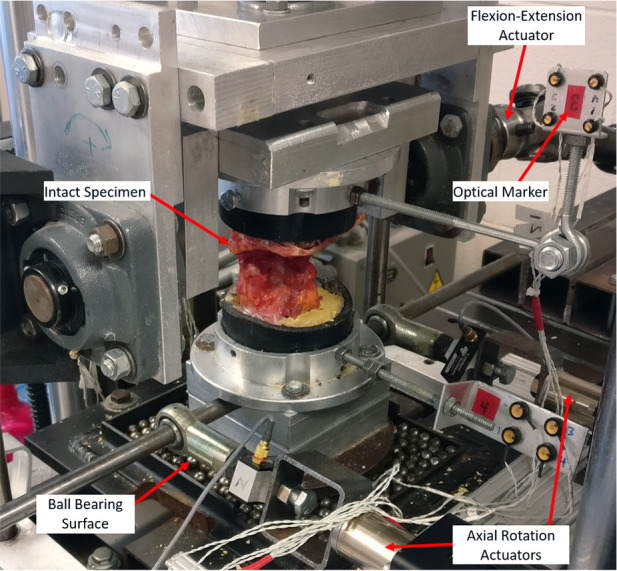
Custom spinal loading simulator used for cadaveric testing.

On the testing dates, the C0 and C3 segment of each specimen was potted in cylindrical molds using dental cement (Heraeus Kulzer, South Bend IN). During the tests, the specimens were instrumented using standard surgical techniques for the C1 lateral masses and C2 pedicles or laminae. The specimens were then mounted in a custom spinal loading simulator^17,18^ (Figure 2) and a previously reported testing protocol was used for each specimen.^19^ Displacement controlled motion was applied in each direction at a rate of 2 deg/s until a load limit of 1.5 Nm was reached with a sampling frequency of 1024 Hz. No axial compression, beyond the weight of the specimen itself, was applied. Motion data of the cadaveric specimens were captured with a sampling frequency of 32 Hz using an optical tracker system (Certus, Northern Digital, Waterloo ON). These motion data were used to report C1-C2 relative motion at an applied load of 1.5 Nm. For each test case, three preconditioning cycles were used^20^ and data were reported for the fourth cycle. The ROM for each motion type was calculated by averaging the motion captured at 1.5 Nm in each direction. Throughout testing, the specimens were kept moist with 0.9% saline (NaCl) solution and the duration of testing was less than 10 h for all specimens.

**Figure 3.**
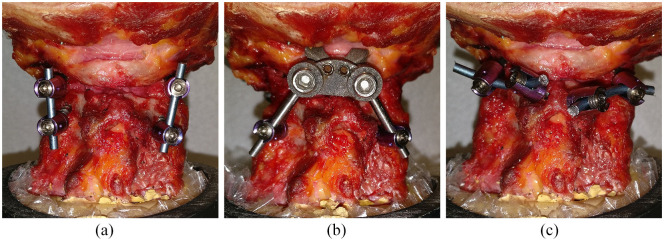
(a) C1LM-C2P, (b) C1PA-C2P and (c) C1LM-C2TL constructs implanted in cadaveric specimens (C1PA-C2TL shown previously in Figure 1(c) and (d)).

**Figure 4.**
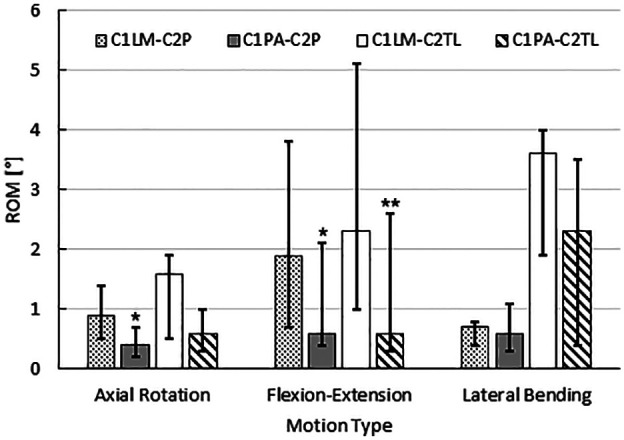
Construct stability at 1.5 Nm as tested in this cadaveric study. Note that * indicates statistically significant difference compared with C1LM-C2P, ** indicates statistically significant difference when compared with C1LM-C2TL.

During the tests, it was intended that each cadaveric specimen would have the motions of right/left axial rotation, flexion-extension and right-left lateral bending applied for each of the following six conditions: (1) intact specimen, (2) destabilized specimen, (3) C1LM screws with C2P screws as described by Harms and Melcher^10^ (Figure 3(a)), (4) C1LM screws with C2TL screws as described by Wright^11^ (Figure 3(c)), (5) C1PA clamp with C2P screws (Figure 3(b)) and (6) C1PA clamp with C2TL screws (Figure 1(c) and (d)). However, two of the cadaver specimens had C2 laminae that were too thin for screw placement and so these two specimens were only tested under four conditions (omitting conditions with C2TL screws). Thus, for constructs that used C2TL screws the sample size was reduced from 8 to 6. The destabilized case was created by resecting the odontoid with a high-speed burr to simulate a Type II odontoid fracture. The screw sizes were as follows: C1LM (4.0 mm diameter, 35 mm length), C2P (3.5 mm diameter, 35 mm length) and C2TL (3.5 mm diameter, 24 mm length).

Due to physical constraints of the testing apparatus, axial rotation was limited to a maximum range of motion of ±15° for the intact and destabilized conditions but this maximum rotation was not reached in any of the construct testing cases. Both the testing order of the constructs and the testing order of the motion type were randomized.

### Statistical analysis

Due to small sample size being an inherent limitation of this cadaver study, normal distributions were not assumed for the test cases. As a result, nonparametric Friedman tests were used to determine whether median C1-C2 ROM differed between test cases. Friedman tests were used to analyse C1-C2 ROM between the intact and destabilized cases as well as to analyse C1-C2 ROM between constructs containing C2P screws and between constructs containing C2TL screws. Statistical comparisons were not made between constructs that used C2P screws and those that used C2TL screws because the present investigation was focused on C1 fixation using either a C1PA clamp or lateral mass screws. Also, advantages and disadvantages of using C2TL screws had been previously reported by others such as Dmitriev et al.^21^ In all cases, a confidence level of 95% was used to determine significance and Minitab statistical software (Minitab LLC, State College PA) was used for calculations.

## Results

### Intact versus destabilized state

For flexion-extension and lateral bending, the destabilized case had significantly more motion compared with the intact case (Table 1) and was in general agreement with existing data from the literature.^12,21,22^ When testing axial rotation, every specimen achieved the maximum rotation of 15 degrees that was allowed by the testing apparatus before the load limit of 1.5 Nm was reached. As a result, statistical comparisons could not be made between the intact and destabilized states for axial rotation and this data has been omitted from Table 1.

**Table 1.** ROM data for intact and destabilized cases; median (minimum–maximum).

Motion	Intact	Destabilized	*p*-Value	*n*
Flexion-extension	4.0° (2.5°–9.4°)	7.6° (3.0°–14.5°)	0.03	8
Lateral bending	1.6° (0.7°–4.3°)	5.2° (3.6°–8.0°)	<0.01	8

*n* = number of samples.

### Construct cases

Of the constructs that had C2P screws (Figure 4, Table 2), the C1PA-C2P construct showed a statistically significant reduction in motion compared with the C1LM-C2P construct in axial rotation and flexion-extension. In the case of lateral bending, a statistically significant difference was not detected between the motion of the C1PA-C2P construct and C1LM-C2P construct. Of the constructs that had C2TL screws (Figure 4, Table 3), the C1PA-C2TL construct showed a statistically significant reduction in motion compared with the C1LM-C2P construct in flexion-extension. In the cases of axial rotation and lateral bending, statistically significant differences were not detected between the motion of the C1PA-C2TL construct and the C1LM-C2TL construct.

**Table 2.** C1-C2 ROM data for constructs with C2P screws; median (minimum–maximum).

Motion	C1LM-C2P	C1PA-C2P	*p*-Value	*n*
Axial rotation	0.9° (0.5°–1.4°)	0.4° (0.2°–0.7°)	0.03	8
Flexion-extension	1.9° (0.7°–3.8°)	0.6° (0.4°–2.1°)	<0.01	8
Lateral bending	0.7° (0.4°–0.8°)	0.6° (0.3°–1.1°)	0.29	8

*n* = number of samples.

**Table 3.** C1-C2 ROM data for constructs with C2TL screws; median (minimum to maximum).

Motion	C1LM-C2TL	C1PA-C2TL	*p*-Value	*n*
Axial rotation	1.6° (0.5°–°1.9)	0.6° (0.3°–1.0°)	0.10	6
Flexion-extension	2.3° (1.0°–5.1°)	0.6° (0.3°–2.6°)	0.01	6
Lateral bending	3.6° (1.9°–4.0°)	2.3° (0.4°–3.5°)	0.10	6

*n* = number of samples.

## Discussion

In the current study, using the C1PA clamp in place of C1LM screws provided an instrumented C1-C2 segment with superior overall stability when used with either C2P or C2TL screws. While this improved stability was statistically significant in flexion-extension and axial rotation for constructs that used C2P screws and in flexion-extension for constructs that used C2TL screws, the magnitude of the difference was quite small and would likely not make a clinical difference to long term fusion rates. However, the novel C1PA clamp was intended to reduce the intraoperative risks and technical challenges of posterior instrumented fusion, many of which stemmed from the use of C1LM screws.

The reduced C1-C2 ROM resulting from the C1PA clamp was believed to be due mostly to improved fixation at the bone-implant interface. The C1PA clamp would use a large surface area of cortical bone for fixation as opposed to C1 lateral mass screws that were only supported by cortical bone at the screw entry points and rely on cancellous bone for most of the screw fixation. Additionally, C1 lateral mass screws had to extend beyond the posterior arch to allow rods to be easily connected. This created an unsupported cantilever beam that increased the moment acting on the implant-bone interface. This increased loading, along with the minimal cortical bone support, was suspected to result in C1 lateral mass screws having more motion at the bone-implant interface compared with the C1PA clamp. Further studies needed to be conducted to determine if this finding was also supported by data from long-term cyclic loading.

The use of either C1LM screws or the C1PA clamp result in similar moment arms to resist axial rotation. While the fixation points of C1LM screws are farther from the centre of rotation than the C1PA clamp in the coronal plane, the fixation points of the C1PA clamp are farther from the centre of rotation in the sagittal plane, effectively giving both constructs about the same ability to resist axial rotation.

A novel C1 posterior locking plate that screwed into the posterior arch was compared with C1LM screws by Kelly et al.^15^ Their testing protocols were similar to the current study but, in their testing, they did not detect a statistically significant difference between stability of the construct with C1LM screws and the construct with their C1 posterior locking plate. They concluded that their locking plate had ‘the potential to provide acceptable stability with greatly decreased surgical risk’. However, due to variability in the shape of the posterior arch, attachment of the plate with locking screws would be technically demanding in many cases.

In contrast to Olerud’s C1 claw^14^ that used C1-C2 transarticular screws to achieve adequate stability to the C1-C2 segment, the C1PA clamp achieved adequate stability under all modes of motion when used with C2 pedicle screws. The surgical skill required to place the C1 posterior arch clamp would be similar to the skill required to place the C1 claw, but placement of C1-C2 transarticular screws was considered more technically demanding than placement of C2 pedicle screws. As a result, the surgical effort associated with constructs that use the C1 posterior arch clamp would be less technically demanding than the effort associated with constructs that used the C1 claw.

During testing, the C1PA clamp was attached more quickly to the cadaveric specimens compared with C1LM screws and the polyaxial rods allowed the C1PA clamp to be easily paired with C2P or C2TL scews. While installation time was not quantified in this study, it was reasonable to suggest that this time savings would be more prominent in a clinical setting because the time consuming dissection to expose the C1 lateral masses would be avoided with the C1PA clamp. Clinically, the C1PA-C2TL construct might be preferred over the C1PA-C2P construct because less lateral dissection would result in a less invasive surgery and eliminate the risk of the C2 screw injuring the vertebral artery. In a cadaveric biomechanics study using fourteen human cervical specimens (C0-C4), Dmitriev et al.^21^ had shown that C2TL screws provide significantly less stability than C2P screws when used in constructs with C1LM screws. Despite the inferior biomechanics of C2TL screws, Parker et al.^12^ demonstrated that the pseudoarthrosis or screw failure rates requiring revision surgery for C2TL screws were not significantly different than that of C2P screws when used with C1LM screws in constructs intended to result in C1-C2 fusion. As a result, clinical efficacy of the C1PA-C2TL construct would probably be similar to the C1LM-C2TL construct based on the similar biomechanics stability of these two constructs that was reported in the present study.

Also, it was noted that the C1 posterior arch had been used clinically as a fixation location for wiring techniques^23^ and interlaminar clamps^24^ that incorporate structural bone grafts. However, these techniques had the potential to injure the dura or spinal cord.^25^ Additionally, in a cadaveric study by Crawford et al.,^26^ 10 human cervical specimens (C0-C6), were loaded non-destructively with a quasi-static system in order to assess the biomechanics of C1-C2 wiring constructs. Constructs were tested under axial rotation, flexion-extension and lateral bending up to a load of 1.5 Nm. Motion was captured using an optical motion analysis system (Optotrak; Northern Digital, Waterloo, ON) and ROM data at the C1-C2 segment was reported for each construct after the first loading cycle and again after 6000 loading cycles. It was found that wiring constructs only moderately reduced motion at the C1-C2 segment but this reduction was not statistically significant in all cases. All wiring constructs were also found to be susceptible to loosening from fatigue. The poor biomechanical stability of wiring constructs has led to a failure or nonunion rate of 10%–15%, which has discouraged the clinical application of these constructs.^27^ The C1PA clamp was designed to reduce the risk of injury to the vertebral artery, dura and spinal cord and data from the current study shows promising biomechanical stability that might positively correlate to clinical fusion rates. Like wiring techniques and interlaminar clamps, use of the C1PA clamp required an intact C1 posterior arch. Combined type II odontoid fracture and fracture of the posterior arch of C1 had a reported prevalence of 14%^28^ and use of the C1PA clamp would be contraindicated in these cases.

### Study limitations

The use of C2TL screws was not possible in two of the cadaveric specimens because the C2 laminae were too thin for screw placement. This reduced the number of tested specimens to six for instrumented C1-C2 segments with constructs having C2TL screws. This was not considered a major concern because the test protocol^19^ referenced for this study suggested that a minimum of six specimens be used for each construct case. Refreezing of cadaveric specimens was done to allow for all of the dissections to be performed by the spinal orthopaedic surgeons in one day. Tan and Uppuganti^29^ reported that the change in neutral zone and ROM characteristics of cadaveric lumbosacral spines was not significant after two freeze-thaw cycles. The upper cervical spines that were tested in the current study might also tolerate freeze-thaw cycles without much change in flexibility. In any case, the constructs themselves and their bone fixation were responsible for most of the instrumented segment stability and thus any change in spine flexibility would likely not significantly influence the results. Due to the physical limitations of the testing apparatus, the axial rotation ROM values for the intact and destabilized states could not be quantified. This did not present a large problem for the current study since the physiological ROM of these states for the upper cervical spine have been well documented in the literature. Only one size of C1PA clamp was tested but it is expected that more sizes would need to be developed to address the full variance of posterior arch anatomies within the population. Also, the degree of osteoporosis present in the cadavers was unknown because bone density scans were not performed. Thus, although nonparametric analysis using a randomized block design (Friedman Test) allowed instrumented C1-C2 segment stability to be compared for the various constructs, the current study did not explore the relationship between degree of osteoporosis and relative construct stability.

### Future work

This study has demonstrated the short term biomechanical stability performance of C1-C2 segments with two novel constructs, the C1PA-C2TL and C1PA-C2P, in a human cadaveric model. Future pre-clinical investigation is required to reduce the bulk of the implant design, develop different sizes, examine the effect of the degree of osteoporosis on construct stability and determine the fatigue failure limit of both these constructs and their bone-implant interfaces under cyclic loading.

### Clinical significance

The surgical technique of implanting the novel C1PA clamp is expected to be less technically challenging than C1LM screws because no dissection is required around the C2 nerve roots and thus, intraoperative blood loss is expected to be reduced. Not having to manage the blood loss associated with exposing the C1 lateral masses is also expected to reduce the operative time of C1-C2 posterior instrumented fusion. This study acts as the first step in developing the C1PA clamp for clinical use but also adds to the literature that explores the feasibility of using the C1 posterior arch as a fixation location and may be helpful in motivating the design of additional devices.

## Conclusions

A novel C1PA clamp has been proposed for use in constructs for the C1-C2 segment and the instrumented segment biomechanical stability was investigated in a cadaveric study. C2P screw constructs with the proposed C1PA clamp showed a statistically significant reduction in motion for flexion-extension as well as axial rotation and no statistical difference was detected in lateral bending when compared to the existing C1LM-C2P construct that has demonstrated clinical efficacy. C2TL screw constructs that used the C1PA clamp showed a statistically significant reduction in motion in flexion-extension and no statistically significant difference in axial rotation or lateral bending when compared to the existing C1LM-C2TL construct that has demonstrated clinical efficacy. Further development of the novel C1PA clamp may result in a better treatment option for elderly patients suffering from atlantoaxial instability.
